# Herpes Simplex Virus Type 2 Myelitis: Case Report and Review of the Literature

**DOI:** 10.3389/fneur.2017.00199

**Published:** 2017-05-10

**Authors:** Raffaele Nardone, Viviana Versace, Francesco Brigo, Frediano Tezzon, Giulio Zuccoli, Slaven Pikija, Larissa Hauer, Johann Sellner

**Affiliations:** ^1^Department of Neurology, Franz Tappeiner Hospital, Merano, Italy; ^2^Department of Neurology, Christian Doppler Medical Center, Paracelsus Medical University, Salzburg, Austria; ^3^Department of Neurorehabilitation Ospedale di Vipiteno and Research Department for Neurorehabilitation South Tyrol, Bolzano, Italy; ^4^Department of Neuroscience, Biomedicine and Movement, Section of Clinical Neurology, University of Verona, Verona, Italy; ^5^Section of Neuroradiology, University of Pittsburgh School of Medicine, Pittsburgh, PA, USA; ^6^Department of Psychiatry and Psychotherapy, Christian Doppler Medical Center, Paracelsus Medical University, Salzburg, Austria; ^7^Department of Neurology, Klinikum rechts der Isar, Technische Universität München, München, Germany

**Keywords:** infectious myelitis, herpes simplex virus type 2, longitudinally extensive transverse myelitis, myeloradiculitis, treatment, outcome

## Abstract

Non-traumatic myelopathies can result from a wide spectrum of conditions including inflammatory, ischemic, and metabolic disorders. Here, we describe the case of a 60-year old immunocompetent woman who developed acute back pain followed by rapidly ascending flaccid tetraparesis, a C6 sensory level, and sphincter dysfunction within 8 h. Acyclovir and steroids were started on day 2 and herpes simplex virus type 2 (HSV-2) was confirmed by polymerase chain reaction in cerebrospinal fluid. Magnetic resonance imaging revealed a bilateral anterior horn tractopathy expanding from C2 to T2 and cervicothoracic cord swelling. Screening for paraneoplastic antibodies and cancer was negative. Neurophysiology aided in the work-up by corroborating root involvement. Recovery was poor despite early initiation of antiviral treatment, adjuvant anti-inflammatory therapy, and neurorehabilitation efforts. The clinical course, bilateral affection of the anterior horns, and early focal atrophy on follow-up magnetic resonance imaging take a necrotizing myelitis potentially caused by intraneuronal spread of the virus into consideration. Further, we summarize the literature on classical and rare presentations of HSV-2 myeloradiculitis in non-immunocompromised patients and raise awareness for the limited treatment options for a condition with frequent devastating outcome.

## Introduction

Longitudinally extensive transverse myelopathy (LETM) describes the condition of a hyperintense spinal cord lesion extending over three or more vertebral levels on sagittal T2-weighted magnetic resonance imaging (MRI). While neuromyelitis optica spectrum disorder (NMOSD) is among the most frequent causes worldwide, a number of other disorders can manifest as or develop LETM over time and have risk of recurrence (1). Thus, a timely diagnosis is driven by the efforts to provide early and appropriate treatment, set measures to prevent future attacks, and avoid severe disability (2). In this regard, the differential diagnosis of LETM beyond NMOSD included multiple sclerosis, acute disseminated encephalomyelitis, parainfectious disorder, subacute combined degeneration, tuberculous myelitis, spinal arteriovenous malformation, and systemic lupus erythematosus in a recent study (3).

Herpes simplex virus type 2 (HSV-2) is a neurotropic virus which can cause genital herpes, aseptic meningitis, encephalitis and myelitis, and devastating infections of the neonate. The belief that the virus reactivates in lumbosacral ganglia is challenged by animal experiments, which found that autonomic ganglia and the spinal cord could be another latency site. Moreover, Ohashi and coworkers showed that HSV-2 reaches dorsal root ganglia and spinal cord independently (4). Myelitis related to HSV-2 is a rare entity and mostly reported in patients with malignancy and acquired immune deficits (5). The classical course of HSV-2 myelitis is ascending myelopathy with subacute development of cervicothoracic sensorimotor impairment. However, fulminant and less rapidly progressing cases have been reported as well (6).

## Case Report

**Figure 1:**
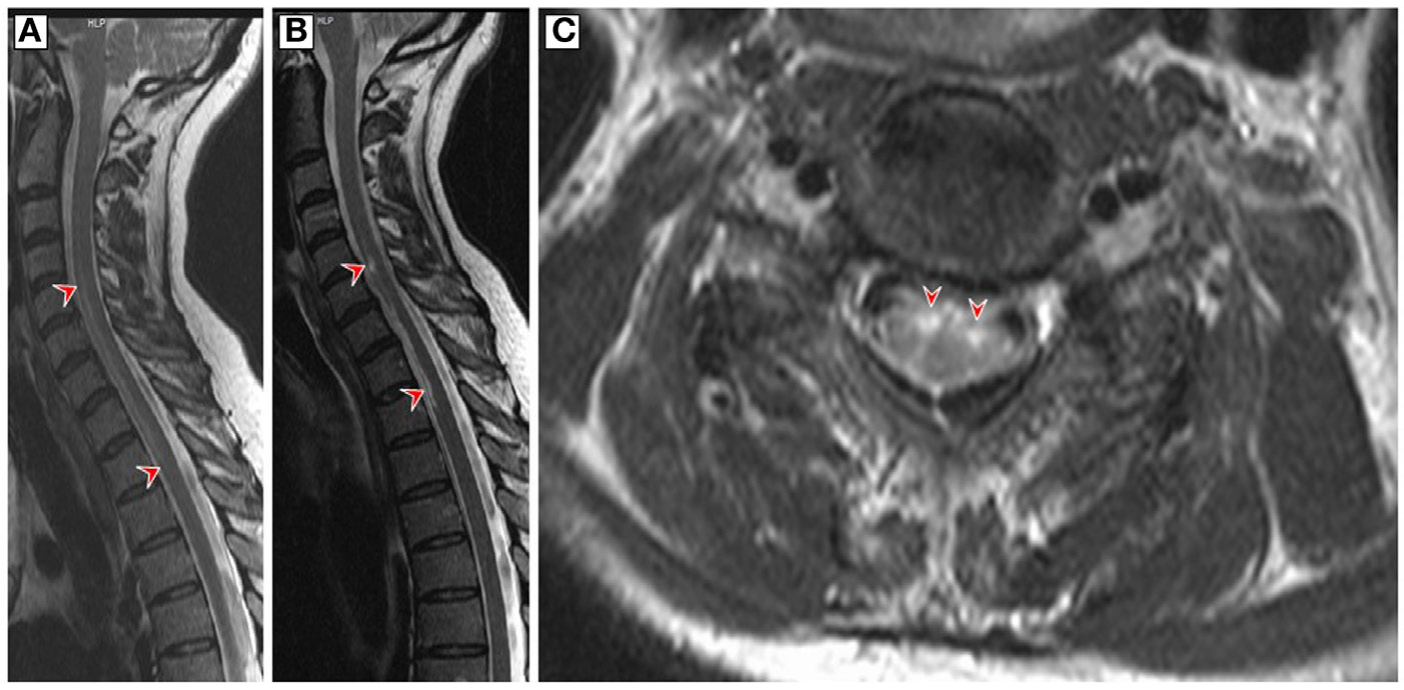
**(A)** Sagittal T2-weighted magnetic resonance imaging (MRI) of the cervicothoracic spinal cord on admission showed a hyperintense “pencil-like” lesion at the level of the anterior horn which extends from the level of the second cervical to second thoracic vertebral level (red arrowheads) and cord swelling in large parts of the affected area. **(B,C)** Follow-up T2-weighted MRI 9 weeks later revealed a less homogenous hyperintense filiform signal in the anterior region of the cervicothoracic cord and circumscribed medullar atrophy in the sagittal plane (red arrowheads). The axial MRI is at the C5 level and demonstrates bilateral hyperintensive signals involving the anterior horns of both sides (owl’s eye appearance, red arrowheads).

A 60-year-old woman developed acute back pain and urinary retention. Ascending sensorimotor disturbances developed thereafter and reached a plateau after 8 h. The neurological examination revealed severe tetraparesis and a sensory C6 level. Deep tendon reflexes were absent in the upper limbs and plantar responses were both extensor. The urodynamic study showed atonic contraction. Spinal cord MRI demonstrated a “pencil-like” T2 lesion expanding from C2 to T2 in the anterior part of the sagittal plane and swelling of the cervicothoracic cord (Figure 1A). Follow-up examination continued to demonstrate a cervicothoracic fusiform lesion in addition to focal atrophy and cavitation (Figures 1B,C). Brain MRI was unremarkable. Cerebrospinal fluid (CSF) analysis showed a normal cell count (2 cells/ml), elevated protein (76 mg/dl), normal glucose levels (65 mg/dl), and IgG index 0.49. Bacterial and fungal cultures were negative, and CSF-specific oligoclonal bands were absent. The test for anti-HSV antibody (ELISA) in the CSF showed positive IgM-HSV (2,000 mg/dl) and negative IgG-HSV. HSV-2 DNA was detected by polymerase chain reaction (PCR) in CSF (50 copies/μl). Other PCR examinations including varicella-zoster virus and enterovirus were negative. Dermatological manifestations did not occur during the entire course. Blood testing for antibodies against aquaporin-4, antinuclear antibodies, anti-neutrophil cytoplasmic antibodies, paraneoplastic antibodies, and HIV were negative. Further unremarkable blood tests included serum protein electrophoresis, angiotensin-converting enzyme vitamin B12, and erythrocyte sedimentation rate. Cancer was ruled out by appropriate measures.

Treatment with intravenous acyclovir (10 mg/kg body weight three times daily) was started on the second day of admission, 4 days in conjunction with betamethasone (8 mg/day). Compound action potentials (cMAPs) and nerve motor conduction velocity were within normal limits on day 2. F-waves were absent in all tested nerves of upper limb and lower limbs, whereas the sensory nerve conduction study was normal. This finding suggested a damage to the anterior horn motor neurons and ventral root fibers. Ten weeks later, neurography showed a severe and inhomogeneous amplitude reduction and absence of cMAPs with relatively preserved conduction velocity in all tested nerves as well as the appearance in some nerves of impersistent and low-voltage F-responses. This confirmed secondary axonal loss in all motor nerves. Electromyography revealed a subacute neuropathic pattern with widespread denervation, minimally altered motor unit action potentials (MUAPs) morphology, and decreased recruitment in the upper limb muscles with different degree of severity. In the examined muscles of the lower limbs, spontaneous activity was less evident and MUAPs recruitment was absent, thus reflecting a reduced central drive.

The neurological examination 3 months later revealed a moderate degree flaccid weakness of the upper limbs, a severe spastic paraparesis and a distended bladder. Now, a sensory level was not evident.

## Discussion

Infratentorial, spinal cord, and peripheral nervous system manifestations of HSV-2 are a relative rarity (7). A Pubmed-search for PCR-confirmed HSV-2 myelitis in non-immunosuppressed patients revealed 11 cases with varying clinical presentation, MRI and CSF findings, dermatological manifestations, chances for recurrence, and persistent neurological deficits (Table 1). Acute, subacute, and ascending progressive courses were present, the ascending necrotizing form is presumably the most devastating subtype (8). Most patients received steroids with varying duration and success on outcome. Yet, some authors assume that this treatment is the mainstay for attenuating immune-mediated damage and halting further disease progression (9). This hypothesis goes back to the observation by Klastersky who reported not only large necrotic areas in the anterior horn and posterior fiber tracts but also multiple demyelinated areas in the white matter in an autopsy case of HSV-2 myelitis (10). The literature search for PCR-confirmed HSV-2 myelitis cases revealed that spinal cord lesions are more frequently located within the thoracolumbar section, and genital herpetic skin lesion are found in a few. A report of a patient series with mostly autopsy-confirmed cases corroborated a rare occurrence of a rash and further disclosed that fever is rare during both onset and progression of the disease (6). Cervical MRI lesions have also been reported with HSV-2 and occasionally in form of LETM. However, anterior horn tractopathy as seen in our case has not been observed among the published HSV-2 cases so far. In contrast, a pediatric case with polio-like presentation caused by HSV-1 was published in 1993 (11). HSV-2 myelitis is assumed to result from virus reactivation, which was latent in dorsal root ganglia (12). It is tempting to speculate intraneural spread of HSV-2 along anterior horn over a large area of the cervicothoracic cord may have been involved in the pathophysiology of our case. The subsequent focal spinal cord degeneration despite early initiation of antiviral and anti-inflammatory therapy is impressive and calls for more research in this condition to examine the pathophysiological basis and treatment options.

**Table 1:** **Herpes simplex virus type 2 (HSV-2) myelitis in non-immunocompromised patients: overview of cases studied by polymerase chain reaction (PCR) of cerebrospinal fluid (CSF) specimen**.

Case no.	Reference	Demographics (gender, years of age)	Comorbidity	HSV-2 PCR in CSF (copies/μl)	Dynamics and clinical syndrome	Additional manifestations	CSF (cells/μl, protein mg/dl)	Longitudinal and axial magnetic resonance imaging lesion expansion	Herpetic skin lesions	Treatment	Outcome at discharge
1	(8)	Female, 76	n.r.	Positive	Subacute, paraplegia, bladder dysfunction, T10 level	Radiculitis	73, 132	T10-conus, enhancement of meninges and roots	Buttocks, tights, abdomen	Acyclovir	Died 21 days from admission
2	(13)	Female, 49	n.r.	Positive	Acute (sudden), paraplegia, back pain, urinary retention, T5/T7 level	Relapse of myelitis	30, 79	C2, posterior (at relapse)	No	Acyclovir, steroids	Complete recovery
3	(13)	Male, 38	n.r.	Positive	Subacute (1 month), paraparesis, bladder dysfunction, T6 level	Relapse of myelitis	11, 75	Normal at relapse	Genital at relapse	Acyclovir, steroids	Paraparesis
4	(9)	Male, 44	Diabetes	Positive	Acute (1 week), paraparesis, urinary problems, T4 level		105, 122	Not performed	Genital	Acyclovir on day 5, steroids	Paraplegia
5	(9)	Male, 69	n.r.	Positive	Acute (1 week), paraparesis, urinary problems, T3 level	Encephalitis	52, 72	T7–L	No	Acyclovir on day 5, vidarabine, steroids, IVIG	Tetraplegia
6	(9)	Female, 50	n.r.	Positive	Acute (1 week), paraparesis, urinary problems, T5 level		39, 51	T7–L	No	acyclovir on day 7, vidarabine, steroids	Paraplegia
7	(9)	Female, 50	n.r.	Positive	Acute (2 weeks), paraparesis, urinary problems, T4 level		158, 99	C–L	No	Acyclovir on day 12, steroids	Paraplegia
8	(9)	Male, 68	n.r.	Positive	Acute (2 weeks), paraparesis, urinary problems, T10 level		3, 51	T5–9	No	Acyclovir after 3 months, steroids	Recovery
9	(9)	Male, 38	n.r.	Positive	Subacute (3 months), paresis of left lower limb, urinary problems, T7 level	Relapse of myelitis	11, 75	Not performed	Genital	Acyclovir after 4 months, steroid	Paraplegia
10	(9)	Female, 49	n.r.	Positive	Subacute (4 months), paresis of right lower limb, urinary problems, T6 level	Relapse of myelitis	196, 90	C2–4, posterior funiculus	No	Acyclovir on day 7, steroids	Recovery
11	(14)	Female, 70	Normal	Negative (positive from gluteal skin lesion)	Acute (couple of days),	3 myelitis episodes, radiculitis	79, 43	Conus medullaris T11–L1, fusiform lesion, myelomeningeal enhancement and posterior nerve roots	Anogenitial and gluteal	Acyclovir on day 1, steroids	Slight proximal paraparesis
12	Current case	Female, 60	Normal	50	Acute (8 h) severe sensomotoric, C6 level	Radiculitis	2, 76	T2 lesion from C2–T2, bilateral anterior horns	No	Acyclovir, steroids	Moderate paresis upper limbs, severe paraparesis lower limbs

*C, cervical; T, thoracal; L, lumbar; n.r., not reported*.

A more stroke-like presentation as in our case was reported for one other report (case #2). Indeed, our case does share some clinical and radiological features of spinal cord infarction (15). The extent of longitudinal expansion, however, does not fit into the concept for vascular supply of the spinal cord and is not reported in the most comprehensive prospective study on spinal cord infarction (16). HSV-2 can cause cerebral ischemia and hemorrhage due to vasculitis (17). As ischemic necrosis is a known neuropathological feature of HSV-2 myelitis, an involvement of vasculitis could be translated to the pathogenesis of the disease (10). It needs to be stressed that most tractopathies are seen with parainfectious or paraneoplastic disorders, or infections, such as poliomyelitis, West Nile virus encephalomyelitis, and tick-borne encephalitis (18–20). All these conditions were ruled out. Moreover, neurophysiology is crucial for identifying, quantifying, and monitoring radicular involvement. A contrast-enhanced spinal MRI, however, would have been ideal to further characterize the current case and exclude additional differential diagnoses, including primary CNS vasculitis of the spinal cord (21).

There are efforts to rationalize the PCR testing for HSV based on CSF cell count and protein levels. Our patient had a normal cell count but elevated protein in CSF and met the Hansen criteria with demand elevated CSF leukocyte count (>5/μl) and/or protein levels (>50 mg/dl) (22). The exception to this guidance includes patient’s age (<2 years) and altered host immune status. PCR results need to be interpreted in the context of the patient’s clinical presentation and timing of the CSF sampling (23). Indeed, the low viral load needs to be seen in the context of early admission, relatively small lesion size, and limited direct CSF contact.

## Conclusion

We report the case of a cervicothoracal tractopathy with stroke-like presentation caused by HSV-2. Outcome was poor despite early antiviral and anti-inflammatory treatment. We conclude that there is need for further neuropathological characterization of the condition and international prospective patient registries, ideally including multimodal treatment concepts.

## Ethics Statement

Ethical approval or patient consent is not required according to national guidelines.

## Author Contributions

RN performed the drafting/revising of the manuscript and accepted responsibility for conduct of research and final approval. VV, FB, FT, GZ, and SP performed the revising of manuscript and acquisition of data and accepted responsibility for conduct of research and final approval. LH and JS performed the drafting/revising of the manuscript and acquisition of data and accepted responsibility for conduct of research and final approval.

## Conflict of Interest Statement

The authors RN, VV, FB, FT, GZ, SP, LH, and JS declare that they have no conflict of interest.

## References

[1] Kister I Johnson E Raz E Babb J Loh J Shepherd TM. Specific MRI findings help distinguish acute transverse myelitis of neuromyelitis optica from spinal cord infarction. Mult Scler Relat Disord (2016) 9:62–7. 10.1016/j.msard.2016.04.005 27645347

[2] Sellner J Boggild M Clanet M Hintzen RQ Illes Z Montalban X EFNS guidelines on diagnosis and management of neuromyelitis optica. Eur J Neurol (2010) 17(8):1019–32. 10.1111/j.1468-1331.2010.03066.x 20528913

[3] Jain RS Kumar S Mathur T Tejwani S. Longitudinally extensive transverse myelitis: a retrospective analysis of sixty-four patients at tertiary care center of North-West India. Clin Neurol Neurosurg (2016) 148:5–12. 10.1016/j.clineuro.2016.06.011 27348743

[4] Ohashi M Bertke AS Patel A Krause PR. Spread of herpes simplex virus to the spinal cord is independent of spread to dorsal root ganglia. J Virol (2011) 85(6):3030–2. 10.1128/JVI.02426-10 21159869 PMC3067948

[5] Widener RW Whitley RJ Herpes simplex virus. Handb Clin Neurol (2014) 123:251–63. 10.1016/B978-0-444-53488-0.00011-0 25015489

[6] Nakajima H Shoji S Herpes simplex myelitis: differences in clinical manifestations between herpes simplex virus type 1 and type 2. In: Hayasaka D, editor. Pathogenesis of Encephalitis. Rijeka: InTech (2011).

[7] Kennedy PG Steiner I. Recent issues in herpes simplex encephalitis. J Neurovirol (2013) 19(4):346–50. 10.1007/s13365-013-0178-6 23775137

[8] Ellie E Rozenberg F Dousset V Beylot-Barry M Herpes simplex virus type 2 ascending myeloradiculitis: MRI findings and rapid diagnosis by the polymerase chain method. J Neurol Neurosurg Psychiatry (1994) 57(7):869–70. 10.1136/jnnp.57.7.869 8021686 PMC1073042

[9] Nakajima H Furutama D Kimura F Shinoda K Ohsawa N Nakagawa T Herpes simplex virus myelitis: clinical manifestations and diagnosis by the polymerase chain reaction method. Eur Neurol (1998) 39(3):163–7. 10.1159/000007927 9605393

[10] Klastersky J Cappel R Snoeck JM Flament J Thiry L Ascending myelitis in association with herpes-simplex virus. N Engl J Med (1972) 287(4):182–4. 10.1056/NEJM197207272870411 4338243

[11] Kyllerman MG Herner S Bergstrom TB Ekholm SE. PCR diagnosis of primary herpesvirus type I in poliomyelitis-like paralysis and respiratory tract disease. Pediatr Neurol (1993) 9(3):227–9. 10.1016/0887-8994(93)90091-P 8394714

[12] Kennedy PG Rovnak J Badani H Cohrs RJ. A comparison of herpes simplex virus type 1 and varicella-zoster virus latency and reactivation. J Gen Virol (2015) 96(Pt 7):1581–602. 10.1099/vir.0.000128 25794504 PMC4635449

[13] Nakajima H Furutama D Kimura F Shinoda K Nakagawa T Shimizu A Herpes simplex virus type 2 infections presenting as brainstem encephalitis and recurrent myelitis. Intern Med (1995) 34(9):839–42. 10.2169/internalmedicine.34.839 8580553

[14] Gobbi C Tosi C Stadler C Merenda C Bernasconi E Recurrent myelitis associated with herpes simplex virus type 2. Eur Neurol (2001) 46(4):215–8. 10.1159/000050808 11721130

[15] Pikija S Mutzenbach J Kunz A Nardone R Leis S Deak I Delayed hospital presentation and neuroimaging in non-surgical spinal cord infarction. Front Neurol (2017) 8:143 10.3389/fneur.2017.00143 28446898 PMC5388752

[16] Masson C Pruvo JP Meder JF Cordonnier C Touze E De La Sayette V Spinal cord infarction: clinical and magnetic resonance imaging findings and short term outcome. J Neurol Neurosurg Psychiatry (2004) 75(10):1431–5. 10.1136/jnnp.2003.031724 15377691 PMC1738740

[17] Zepper P Wunderlich S Forschler A Nadas K Hemmer B Sellner J Pearls & Oy-sters: cerebral HSV-2 vasculitis presenting as hemorrhagic stroke followed by multifocal ischemia. Neurology (2012) 78(3):e12–5. 10.1212/WNL.0b013e31823fcd4d 22249502

[18] Steiner I Kennedy PG. Acute disseminated encephalomyelitis: current knowledge and open questions. J Neurovirol (2015) 21(5):473–9. 10.1007/s13365-015-0353-z 26037112 PMC7095407

[19] Sejvar JJ. West Nile virus infection. Microbiol Spectr (2016) 4(3). 10.1128/microbiolspec.EI10-0021-2016 27337465

[20] Pichler A Sellner J Harutyunyan G Sonnleitner A Klobassa DS Archelos-Garcia JJ Magnetic resonance imaging and clinical findings in adults with tick-borne encephalitis. J Neurol Sci (2017) 375:266–9. 10.1016/j.jns.2017.02.003 28320144

[21] Salvarani C Brown RD Jr Calamia KT Christianson TJ Huston J III Meschia JF Primary CNS vasculitis with spinal cord involvement. Neurology (2008) 70(24 Pt 2):2394–400. 10.1212/01.wnl.0000314687.69681.24 18541872

[22] Hanson KE Alexander BD Woods C Petti C Reller LB. Validation of laboratory screening criteria for herpes simplex virus testing of cerebrospinal fluid. J Clin Microbiol (2007) 45(3):721–4. 10.1128/JCM.01950-06 17202281 PMC1829123

[23] Tyler KL. Herpes simplex virus infections of the central nervous system: encephalitis and meningitis, including Mollaret’s. Herpes (2004) 11(Suppl 2):57A–64A. 15319091

